# Hematopoietic Cell Transplantation for Older Patients with MDS

**DOI:** 10.4084/MJHID.2014.056

**Published:** 2014-09-01

**Authors:** Mazyar Shadman, H. Joachim Deeg

**Affiliations:** Fred Hutchinson Cancer Research Center and the University of Washington School of Medicine, Seattle, WA, USA, 98109-1024.

## Abstract

The incidence of myeloid malignancies, including myelodysplastic syndromes (MDS) increases with age. While several therapeutic modalities have been developed, for most of these patients the only treatment with curative potential is allogeneic hematopoietic cell transplantation (HCT). The development of reduced/low intensity transplant conditioning regimens allows to successfully transplant patients in their ‘60s and even ‘70s, although comorbidities may determine who does come to transplantation and who does not. Also, as many as half of the patients will develop graft versus host disease (GVHD), even with HLA matched donors, requiring therapy for extended periods of time, and GVHD and treatment with glucocorticoids is likely to impact the quality of life. Nevertheless, dependent upon disease stage at HCT, the presence of comorbidities and the regimen used, 30% to 50% of patients 60 years of age or older, may survive long-term cured of their disease. Future studies should focus on the incorporation of non-transplant modalities into the overall transplant approach, the prevention of GVHD, and the utilization of immunotherapy to reduce the incidence of relapse and GVHD and further improve overall transplant success.

## Introduction

The past decade has seen explosive developments of our understanding of the genetics and molecular biology of myeloid disorders, and new classification schemes incorporating those insights are emerging. The development of drugs exploiting the improved understanding of disease mechanisms is changing therapeutic strategies and altering the natural disease course. While those strategies for myelodysplastic syndrome (MDS) or myeloproliferative neoplasms (MPN) are not curative, they impact the decision regarding allogeneic hematopoietic cell transplantation (HCT), particularly in older individuals.

Nevertheless, allogeneic HCT is currently the only modality with proven curative potential. As increasing age is associated with a greater prevalence of comorbidities, HCT had been reserved, until recently, for younger individuals. The development of novel strategies now allows to transplant, successfully, patients in the seventh or even eighth decade of life. However, HCT is not without potential problems including post-transplant relapse, and graft-vs.-host disease (GVHD). These issues raise ethical questions, particularly in regards to *quality* of life (QOL) vs. *quantity,* and with the availability of non-transplant treatment options that modify the natural disease course, patients (or physicians) may prefer treatment, for example, with hypomethylating agents, rather than proceeding to HCT*.* There are also socioeconomic issues and questions of health resource utilization. Both transplant and non-transplant modalities are cost-intensive, and treatment cost and insurance coverage are issues that need to be addressed. We will review here some aspects of HCT in older individuals with a focus on MDS.

## Background

The spectrum of MDS ranges from very low-grade disease with life expectancies of a decade or two, to aggressive disease with a life expectancy of only a few months, typically related to progression to acute myeloid leukemia (AML).^1^ However, in all patients, MDS is a clonal disease of hematopoiesis, and what determines slow progression of marrow failure (without evolution to leukemia) in some patients, and rapid progression to AML in others is only beginning to be understood. More than forty somatic mutations in hematopoietic cells have been described by now, and there is strong evidence that certain mutations, for example, in ASXL1, RUNX1, SRSF2 or TP53, are associated with a more rapid progression of the disease. Conversely, other mutations, for example, in TET2, may be associated with slower disease evolution than observed with wild-type TET2.^2^,^3^ Available data also suggest that mutations in genes of the splicing machinery, occurring early in the disease course, are the strongest “driver mutations”, and that mutations, for example, in histone or DNA-modifying genes, in transcription factors, such as TP53, and in kinase genes follow later.^4^ One clone may be dominant early in the disease, but follow-up, as described by Walter et al., will show the emergence of sub-clones with new mutations, which may convey an altered prognosis.^5^,^6^ These observations are important not only because of the underlying disease mechanism and pathophysiology, but also because a given treatment may prove to be more efficacious in the setting of one mutation than in another and, in fact, may lead to the selection of more resistant clones. These patterns are, of course, not unique to MDS or AML; intra-tumor heterogeneity has been observed in many malignancies.^7^

In addition to somatic mutations, numerous single nucleotide polymorphisms (SNP) have been described that may affect the disease process and may serve as predisposing factors for the development of clonal disorders such as MDS.^8^,^9^ Further, recent data indicate that even in the absence of mutations, dysregulation of expression of various transcription factors, for example, TWIST1, and of microRNAs (miRs) may be instrumental in disease evolution.^10^–^12^ In fact, it has been suggested that miRs are the final regulators of tumor progression.^13^ While this concept has added complexity to our understanding of disease mechanisms, it is expected that these insights will also lead to the development of novel therapies utilizing recombinant RNA/DNA technology. While such therapy is currently “not ready for prime time”, investigations are ongoing that use approaches based on findings such as spliceosome mutations or miR dysregulation.

## Current non-Tranplant Therapy for MDS

It is agreed upon that patients with a deletion of the long arm of chromosome 5, del(5q), if they do have peripheral blood cytopenias, in particular, anemias, should be treated with lenalidomide (Revlimid).^14^,^15^ The probability of becoming transfusion independent is about 65%, and many patients normalize their marrow, including cytogenetics. On average transfusion independence extends over 2 to 2.5 years. However, even this remarkable treatment does not eradicate the clone, which persists in stem cells.^16^

For other MDS patients, hypomethylating agents (HMA), i.e. 5-azacitidine (5-aza) or 2-deoxy-5-azacitidine, are standard therapy, certainly for patients in IPSS categories intermediate-2 and high, although patients with lower risk disease may benefit, by achieving improved blood cell counts.^17^–^19^ Approximately 40% to 50% of patients will have hematologic responses, leading to improved QOL. However, on average these responses are sustained for only 9 to 10 months, notwithstanding the fact that some patients may benefit for years. Numerous other agents or combination therapy, for example, lenalidomide combined with HMA, hold promise.^20^ However, none of these approaches is curative. This raises several questions. If a patient is interested in a curative approach, is initial treatment with HMA (or lenalidomide) impacting the success of transplantation? If so, in a positive or negative way? What should be the timing of HCT in a patient who is responding to HMA? Has the patients acquired additional comorbidities while receiving treatment with HMA (or other agents), while reflecting on the possibility of transplantation? And how might those comorbidities affect the decision for or against transplantation and the success rate of HCT?

## Complicating Factors for Transplantation in Older Patients

At the time of diagnosis of MDS, patients are, on average, 70 to 75 years old. Some 10 or 15 years ago, one would not have considered HCT for patients in this age range. A review of the literature shows, however, that the median age of transplanted patients has increased continuously over the past few decades. The database of the Center for International Blood and Marrow Transplantation Research (CIBMTR) shows a median patient age of 25 years in the 1980s, 39 in the 1990s, and 46 years over the past decade. Among patients transplanted between 2002 and 2009, 44% were older than 50 years, and the proportion of patients older than 60 years increased from 8% to 17% between 2002 and 2011.^21^

### Why the earlier reluctance and why the current willingness to transplant older patients?

The often very intensive conditioning regimens used historically were poorly tolerated by older individuals, presumably related to “biologic age”. However, it is clear from recent studies that it is not so much chronological age but, rather, medical issues, co-morbid conditions, more likely to be present with advanced age that negatively impact outcome. Several scoring systems to assess comorbidities and other factors that may affect transplant outcome have been developed. Parimon et al.^22^ presented a predictive index referred to as pre-transplantation Assessment of Mortality (PAM), based primarily on donor type, disease risk, conditioning regimen, FEV_1_, carbon monoxide diffusing capacity, serum creatinine, and serum alanine aminotransferase concentrations, as well as age. Most of the studies using the PAM have been carried out in patients transplanted following high-intensity conditioning regimens, i.e. generally younger patients and patients without comorbidities; a clear assessment and validation of this test for older patients or patients conditioned with reduced-intensity conditioning regimens remains to be done. Sorror and colleagues developed the Hematopoietic Cell Transplantation Comorbidity Index (HCT-CI), which considers only patient conditions, not other factors that are part of the PAM.^23^ The HCT-CI includes various cardiac, metabolic, cerebrovascular and hepatic parameters, but also rheumatologic disorders, in addition to pulmonary dysfunction, psychiatric disorders, and a prior history of solid tumors. This scoring system has been applied to patients conditioned with reduced-intensity “nonmyeloablative” conditioning regimens, and a clear inverse correlation between the HCT-CI score and transplant outcome has been shown.^24^

Most recently, Della Porta and colleagues presented data specifically for patients with MDS whom they classified by using the revised IPSS ( IPSS-R), the HCT-CI (low/intermediate or high), the presence of monosomal karyotype, refractoriness to induction chemotherapy, and patient age (less than or greater than 50 years) for an MDS transplantation risk index [TRI]).^25^ Their data on 519 patients suggest that all these factors impact transplant outcome. Specifically, the probability of long-term survival with a score of 0 or 1 was 70%, whereas there was a median survival of only a few months (and no long-term survival) among patients with a score of > 4, which could be reached, for example, with a high-risk HCT-CI, a high-risk IPSS-R, and age > 50 years.

Of course, as shown by Naqui et al., comorbidities in patients with MDS also impact negatively survival in non-transplanted patients.^26^ In other words, patients who would benefit most from definitive therapy with HCT, are also the highest-risk patients when transplanted. Therefore, detailed discussions with the patient in preparation for transplantation are essential.

Koreth et al. analyzed data on 514 patients with de novo MDS, 60 to 70 years of age, and compared results obtained with HCT following reduced-intensity conditioning (RIC) to results with best supportive care (growth factor therapy or hypomethylating agents in patients with intermediate-2/high-risk disease by IPSS).^27^ Patients with low/intermediate-1 risk IPSS, had a life expectancy after transplantation of 38 months, compared to 77 months for patients who were not transplanted. A quality-adjusted life expectancy (QALE) and sensitivity analysis did not favor the use of HCT in those patients. Conversely, patients in intermediate-2/high-risk IPSS the life expectancy was 36 months for transplanted, compared to 28 months for non-transplanted patients, thereby favoring HCT. However, patients had to survive for 3 – 4 years before this advantage of HCT became apparent.

## What can an Older Patient Expect from Transplantation?

There are no randomized, prospective studies comparing HCT with non-transplant results in any population of patients, including patients older than 60 or 70 years. Two such studies are currently underway in Europe and the United States.

Published retrospective data have compared results in older and younger patients with comparable diagnoses. For example, Sorror et al. provide an analysis of transplant results in patients with various diagnoses who were 60 to 64, 65 to 69, or 70 years of age and older, and suggested that, based on likelihood ratio statistics using a Cox regression model, there was no significant difference in survival between those age brackets (*P* = 0.18).^28^ Similar data have been presented by others (see below). However, those results have to be viewed critically. While statistical adjustments can be made in the analysis, there is agreement that older patients were selected for transplantation because they were considered *“good candidates”,* i.e. with no or few comorbidities, appearing younger than their stated age with a high level of motivation and commitment. These patients, therefore, do not necessarily reflect the average patient in that age bracket in whom the diagnosis of a malignancy amenable to transplantation is being made.

The Minnesota team analyzed results in 124 MDS (28%) and AML (72%) patients 55–70 years of age who were conditioned with non-myeloablative regimens and received transplants from HLA- matched related donors (MRD) or were given umbilical cord blood (UCB).^29^ The overall survival at 3 years was 37% for MRD and 31% for UCB transplants, respectively. Acute (grades II–IV) and chronic GVHD rates were 38%–45% and 26%–33% for MRD and UCB donors, respectively, and treatment–related mortality at 2 years was 25%.

The European Bone Marrow Transplantation (EBMT) group reported results in 1,333 patients who were 50 to 74 years old at the time of HCT and carried the diagnosis of MDS or secondary AML.^30^ They were transplanted from HLA-identical siblings (*n* = 811) or from unrelated donors (*n* = 522). In this cohort, 883 patients were 50 to 60 years of age, and 449 were older than 60 years. In this analysis, age was not a significant risk factor for outcome. Relapse was determined by advanced disease stage (*P* < 0.01), and RIC regimen (*P* < 0.01). Non-relapse mortality was determined by disease stage (*P* = 0.01), the use of unrelated donors (*P* = 0.03), and the use of RIC regimens (*P* = 0.03). Thus, these data suggest that the selection of conditioning intensity, which very likely considered patient age, was the most determining factor for outcome.

The CIBMTR presented data on 1,080 patients transplanted between 1995 and 2005 following conditioning with RIC regimens.^31^ Among patients with MDS or AML (in first remission), 2-year survival was 44%, 50%, 34%, and 36% for patients 40 to 54 years of age, 55 to 59 years, 60 to 64 years, or older than 64 years (*P* = 0.05). Cytogenetics impacted relapse and relapse-free survival. The 2-year overall survival was determined by the pre-HCT performance status. Non-relapse mortality was not significantly associated with chronologic age.

It remains challenging to define “biologic age”. Recent efforts have considered frailty as a helpful parameter, suggesting an inverse relationship between frailty and likelihood of success after HCT.^32^

## Who and How?

Thus, the available data indicate that HCT is feasible in older patients with MDS (and other myeloid diseases); however, as emphasized above, no prospective trials are available. Therefore, how should patients be selected and what transplant strategy should be pursued? Clearly, disease and patient-associated risk factors, the tempo of the disease, donor availability, among other factors, need to be considered. Based on the results summarized here, patients with more advanced disease, including IPSS intermediate-2, or high-risk patients or with intermediate to very high-risk in the WPSS classification, and patients in the intermediate to very high-risk categories as determined by the IPSS-R scoring, should be considered for HCT. This does not preclude, of course, transplantation for patients with lower risk under particular circumstances, such as heavy transfusion dependence without significantly elevated myeloblast counts and without high-risk cytogenetics or other cytopenias, which per se would not place a patient in a very high-risk category. We know, however, that in patients with those characteristics the disease tends to progress more rapidly, and carries a higher risk of transform into AML. Further, iron accumulation associated not only with the disease itself but particularly with the heavy transfusion load, may add comorbidity if the transplant is delayed.^33^ Also, patients who by accepted criteria have low-risk disease but have significantly aberrant immunophenotypes of blast cells, as determined by flow cytometry,^34^ should have an opportunity to discuss transplantation early in the course.

Investigators at the MD Anderson Cancer Center proposed several additional risk scores, including a simplified MDS risk score that considered poor performance status, older age, thrombocytopenia, anemia, increased marrow blasts, leukocytosis, and high-risk cytogenetics by IPSS criteria, and transfusion need as adverse risk factors. Several studies have validated this scoring system, and patients who have high scores by this assessment should probably be assessed for transplantation if they are motivated, and the appropriate support is available.^26^,^35^

Generally, patients with an HCT-CI score of > 2 have experienced considerably higher mortality post-transplant than patients with lower scores. For patients with high HCT-CI scores even RIC or “non-myeloablative” regimens as used currently may be associated with unacceptable toxicity.

For patients with advanced disease, the challenge is two-fold: Since older patients will be prepared for transplantation with RIC regimens, providing a less cytotoxic component and a lesser debulking effect than is achievable with high-intensity conditioning regimen, it appears advisable (although no controlled data exist) to use debulking therapy before transplantation. Several retrospective analyses have attempted to determine the impact of pre-transplant therapy, particularly with HMAs. It is premature to draw firm conclusions, again, because no controlled studies are available. Clinical wisdom, however, holds that a debulking attempt is indicated in patients with 5% myeloblasts or more who are heading for a RIC transplant regimen. Classically, induction-type chemotherapy has been used which historically has been associated with mortality in the range of 10% or even higher. The advent of HMAs offers an alternative. They are well tolerated, and 40% to 50% of patients derive clinically relevant responses. Responding patients, however, may be reluctant to proceed to HCT since they are doing well and are not prepared to accept the potential risks associated with HCT, but there are draw-backs.^36^ Prebet et al showed that patients who received 5-azacitidine but their disease progressed while on treatment, had a life expectancy of 5–6 months. HCT was the only modality that offered any hope, but even so, the median survival was only 1 to 1.5 years. On the other hand, patients who were taken off treatment because they did not respond or did not tolerate the drug and, therefore, went to transplantation, had a probability of about 40 % of becoming long-term survivors. Thus, if patients receive HMAs and are interested in and are candidates for HCT, one should, presumably, proceed to HCT while the patient is still responding, assuming a donor is available.

Field et al., in a retrospective analysis, observed overall survival, relapse-free survival, and relapse incidence at 1 year of 47%, 41%, and 20%, respectively, in patients who had received 5-azacitidine, compared to 60%, 51%, and 32%, respectively, in patients who had not received the drug before transplantation.^37^ As in other studies, however, the selection of patients for treatment vs. no treatment was likely to be biased.

One prospective study comparing 5-azacitidine to induction-type chemotherapy in patients with advanced MDS who are candidates for HCT was recently initiated by the FHCRC team with the objectives of determining the response to either strategy, of determining what proportion of patients with either approach could be brought to transplantation, and assessing the impact on post-transplant outcome.^38^

## What is the Optimum Conditioning Regimen?

Even in younger patients the answer to this question is not clear. A randomized trial comparing high-intensity and RIC regimens in patients with MDS or AML conducted in the U.S. under the auspices of the BMT CTN has recently been closed, and data should be forthcoming soon.^39^ We know from retrospective analyses that there are patients more than 60 or even 70 years of age who tolerate high-intensity conditioning regimens as used in younger patients (for example, combinations of busulfan and cyclophosphamide or fludarabine and busulfan).^40^ The general clinical policy is to condition patients older than 60 or 65 years with RIC regimens, although those “RIC” comprises regimens with higher intensity than what is generally referred to as “nonmyeloablative” (such as fludarabine + 2 Gy TBI). Those regimens include combinations of fludarabine + melphalan or fludarabine + reduced-dose busulfan (8–10 mg/kg, which can be administered in the outpatient setting, favored by most patients and, conceivably, has a cost-sparing effect as well.

However, the incidence of disease relapse after RIC in most such studies has been higher than with high-intensity regimens; the incidence of GVHD has been very similar, although the severity may be lessened.^41^ While disease recurrence, observed in as many as 40% of patients with high risk MDS, may return patients to a disease stage not very different from the pre-HCT situation, GVHD clearly induces a new problem, the classic “secondary disease”. Since first-line therapy generally is with glucocorticoids, and older individuals often do not tolerate glucocorticoids well, this scenario may lead to a downward spiral of clinical deterioration. Patients may develop myopathy and become progressively inactive, further enhancing the risk of potentially fatal infections. New strategies are needed.

Very promising results have been obtained in recent years with treosulfan-based regimens used in patients up to 65 years of age.^42^ Treosulfan metabolism differs from that of busulfan and is associated with very little non-hematological toxicity. For patients who do not have high-risk cytogenetics, 2-year relapse-free survival as high as 80% has been reported. Recent evidence suggest that the addition of 2 Gy TBI may improve results for patients with high-risk cytogenetics as well, showing a 2-year relapse-free survival of about 65% (compared to 40% without incorporation of TBI), possibly related to a radiosensitizing effect of treosulfan.^43^

## Which Source of Stem Cells?

In recent years, the use of G-CSF-mobilized peripheral blood progenitor cells (PBPC) has been favored because of more rapid engraftment, i.e. shorter duration of pancytopenia, in particular neutropenia, and a more potent anti-tumor (GVL) effect than observed with marrow cells. However, a randomized study in patients receiving unrelated donor transplants, similar to earlier studies with HLA-identical sibling transplants, showed a higher incidence of chronic GHVD with PBPC, even though this did not impact long-term survival.^44^ However, as stated above, long-term steroid use (for GVHD) will create new problems, particularly in these older patients and will affect the QOL.

Umbilical cord blood and HLA-haploidentical related donors offer additional transplant options, greatly expanding the donor pool such that a suitable donor/ source of stem cells is available for almost all patients.^45^ The use of cord blood cells, in particular, has been associated with relapse rates lower than seen, for example, with cells from HLA matched unrelated donors; the GVHD incidence may not be significantly different from that with HLA-matched donor cells.^46^ Some promising results have been reported also with HLA-haploidentical donors, generally with the use of marrow as a source of stem cells. In fact the incidence of GVHD tends to be lower than observed with HLA-identical transplants, largely due to the effect of post-transplant administration of cyclophosphamide ( days +3 and + 4).^47^

## Summary and Conclusions

There has been considerable progress with the use of allogeneic HCT in general, and in older patients and those with MDS in particular. Regimen-related toxicity has been reduced with the development of a range of different intensity conditioning regimens. Prevention and treatment of GVHD, however, remain challenging tasks. Particularly older patients, in whom GVHD and its treatment with glucocorticoids may have a major impact, need to be fully aware of what they are facing should they develop GVHD.

Nevertheless, all patients with MDS in their 60s or even early to mid-70s should be offered an open discussion regarding an overall treatment plan, including current standard therapy with transfusions, growth factors or HMAs, as well as transplantation and long-term outcome. A stem cell source can now be identified for almost all patients, and the limiting factors in the decision-making process are the patient’s comorbidities and their likely impact on transplant outcome, the patient’s understanding of the procedure and long-term effects, as well as the financial impact, not only on the patients themselves but also on their families and, certainly, the long-term QOL.

Current and future research must further reduce treatment-related morbidity and mortality. The transplant community must do better with incorporating non-transplant modalities into the overall management of patient with MDS, and some of these modalities such as HMAs or immunotherapy must be investigated in well-designed post-transplant studies.

## Figures and Tables

**Figure 1:**
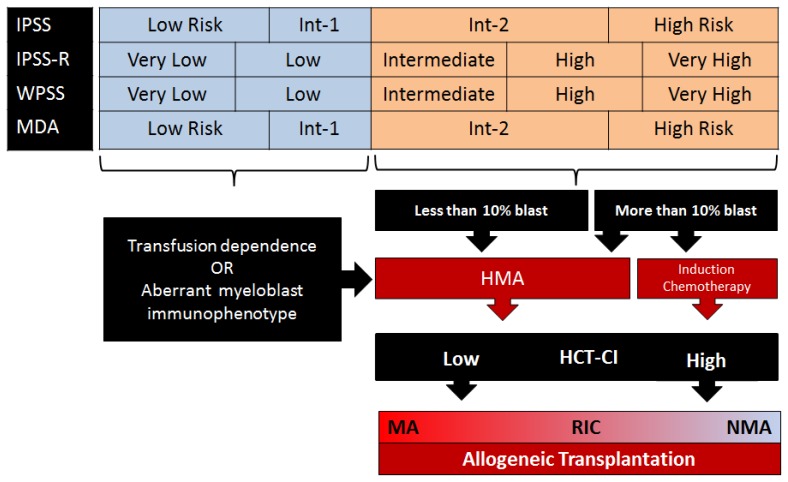
Treatment strategy in older patients with MDS based on available evidence. (IPSS: International Prognostic Scoring System^48^; IPSS-R: Revised International Prognostic Scoring System^1^; WPSS: WHO Classification-Based Prognostic Scoring System^49^; MDA: MD Anderson Prognostic Risk Model^35^; HMA: Hypomethylating agents ;HCT-CI: Sorror Hematopoietic cell transplantation (HCT)-specific comorbidity index^23^,^28^; MA: Myeloablative;RIC: Reduced-intensity ;NMA:Non-myeloablatvie)
